# CHD-CXR: a de-identified publicly available dataset of chest x-ray for congenital heart disease

**DOI:** 10.3389/fcvm.2024.1351965

**Published:** 2024-04-08

**Authors:** Li Zhixin, Luo Gang, Ji Zhixian, Wang Sibao, Pan Silin

**Affiliations:** Heart Center, Women and Children's Hospital, Qingdao University, Qingdao, China

**Keywords:** publicly dataset, congenital heart disease, artificial intelligence, computer vision, cardiovascular health

## Abstract

Congenital heart disease is a prevalent birth defect, accounting for approximately one-third of major birth defects. The challenge lies in early detection, especially in underdeveloped medical regions where a shortage of specialized physicians often leads to oversight. While standardized chest x-rays can assist in diagnosis and treatment, their effectiveness is limited by subtle cardiac manifestations. However, the emergence of deep learning in computer vision has paved the way for detecting subtle changes in chest x-rays, such as lung vessel density, enabling the detection of congenital heart disease in children. This highlights the need for further investigation. The lack of expert-annotated, high-quality medical image datasets hinders the progress of medical image artificial intelligence. In response, we have released a dataset containing 828 DICOM chest x-ray files from children with diagnosed congenital heart disease, alongside corresponding cardiac ultrasound reports. This dataset emphasizes complex structural characteristics, facilitating the transition from machine learning to machine teaching in deep learning. To ascertain the dataset's applicability, we trained a preliminary model and achieved an area under the receiver operating characteristic curve (ROC 0.85). We provide detailed introductions and publicly available datasets at: https://www.kaggle.com/competitions/congenital-heart-disease.

## graphical abstract

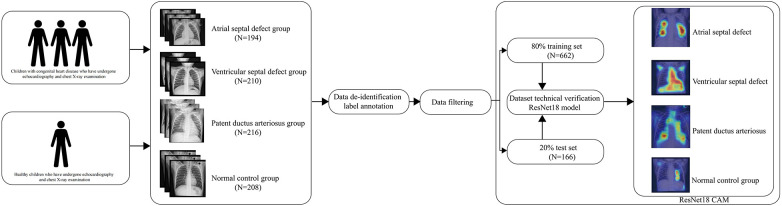

## Introduction

Congenital heart disease accounts for one-third of major birth defects and is the most common heart disease (1, 2). The incidence rate of congenital heart disease in newborns is approximately 0.4%–1.2% (3). Cardiac ultrasound is the main measure for early detection, but it requires specialized cardiac ultrasound doctors (4, 5). In underdeveloped medical areas, congenital heart disease is easily missed. The guidelines point out that standardized chest x-rays can also be used to assist in the diagnosis and treatment of congenital heart disease (5). However, due to the not-so-obvious cardiac manifestations, they have not received sufficient attention. Nevertheless, for cardiac doctors in underdeveloped medical areas, standardized chest x-rays can be used to assist in indicating the occurrence of congenital heart disease. With the support of deep learning in computer vision, it is currently possible to identify large areas of pneumonia and other abnormalities (6). With the advancement of computers, machine learning will transform into machine teaching, artificial intelligence to teach humans more experiences. Computer vision is gradually developing towards fine-grained image analysis (7), allowing for the identification of subtle changes that humans may not notice, such as lung vessel density in chest x-rays of children with congenital heart disease. This information reminds patients that they should promptly seek professional cardiac ultrasound examination in developed medical areas.

The advancement of deep learning is dependent on the availability of high-quality image datasets. Despite the rapid growth in GPU computing power for deep learning, the shortage of expert-annotated high-quality medical image datasets stands as the primary constraint in the progress of medical image AI. Presently, numerous publicly accessible databases focusing on chest x-rays have been released in the field (8–12). Nevertheless, these databases primarily concentrate on macroscopic lesions and pulmonary diseases that are observable to the naked eye, and they have not completely exceeded the diagnostic abilities of clinical physicians. They still operate within the realm of machine learning. The ongoing trajectory in the evolution of medical image AI revolves around utilizing the outstanding observational capacity of machine learning to excel beyond the diagnostic proficiency of clinical practitioners, transitioning from machine learning to machine teaching.

To address this, we have developed and released an expert-annotated high-quality dataset for training fine-grained models in the field of congenital heart disease. The dataset includes 828 DICOM chest x-ray files from children, divided into four categories: atrial septal defect(ASD), ventricular septal defect(VSD), patent ductus arteriosus(PDA), and normal control group. Compared to other published chest x-ray datasets, our dataset focuses more on the identification and classification of fine-grained structural features, promoting the transition of deep learning from machine learning human experience to human learning machine experience.

## Method

The establishment of the dataset is divided into four basic steps. (1) Data collection (2) Data de-identification and labeling annotation (3) Data filtering (4) Technical validation.

### Data collection

Population distribution design and matching were conducted prior to data collection to ensure matching of gender and age distributions among different groups, preventing potential confounding factors. Data collection was carried out at Qingdao Women and Children Hospital from 2021 to 2022. Each chest x-ray corresponding to a specific cardiac condition is accompanied by a cardiac ultrasound report from the respective child, ruling out other cardiac or pulmonary conditions. The cardiac ultrasound reports were evaluated by three professional cardiac ultrasound doctors. Cardiologists summarized information on children with congenital heart disease who received inpatient treatment and regularly conducted follow-up examinations, including chest x-rays and cardiac ultrasounds. The data for children in the healthy control group came from healthy children who had health checks in our hospital. Matching gender and age were performed on children in each group to prevent biases between groups.

The ethical review of this study has obtained approval from the hospital's ethics committee. The time discrepancy between the cardiac ultrasound report and the chest x-ray does not exceed 3 days. The initial storage format of chest x-ray images is DICOM, and the cardiac ultrasound reports were reviewed by three specialized cardiac ultrasound physicians to confirm the diagnosis of congenital heart disease. To facilitate subsequent model training, scale the image to 500 × 500 pixels.

### Data de-identification and label annotation

The acquired chest x-ray DICOM files include sensitive patient information such as age, name, and examination time. To safeguard patient privacy, we developed a de-identification program to convert the DICOM files into a more suitable and commonly used file format (JPG) for deep learning purposes. The cardiac ultrasound reports encompass various sensitive parameters. Three cardiac ultrasound specialists annotated the reports following the diagnostic standards for congenital heart disease outlined by the Chinese Medical Association. These reports were classified into the normal control group, atrial septal defect group, ventricular septal defect group, and patent ductus arteriosus group. Any ultrasound reports that did not achieve consensus among the three cardiac ultrasound specialists were excluded from the dataset.

### Data filtering

Specialized cardiologists conducted data filtering to eliminate confounding factors that could impact the images. These factors encompass additional cardiac complications like Tetralogy of Fallot, pneumonia, tuberculosis, chest wall deformities, pectus carinatum, and pectus excavatum.

### Technical verification

To verify the usability of the dataset in deep learning, we used Pytorch (13) to build a lightweight model for training and preliminary validation on the NVIDIA 4090 graphics card platform. In order to improve the model's ability to recognize local details and extract features, we chose ResNet18 as the base model. The ResNet model can avoid gradient explosion caused by increased depth (14). In order to accelerate model training speed and avoid waste of computational resources, we introduced the idea of transfer learning (15) and pre-trained it using the classic image dataset ImageNet (16). Then we trained on our congenital heart disease dataset, for each evaluation metric, both the overall model performance and the compartment-wise model performance were plotted. In order to reduce overfitting, we introduced randomized tiling, color inversion, and mirroring for data augmentation. In order to determine the credibility and stability of the model and enhance clinicians' confidence in the model, we used CAM(Class Activation Mapping) to analyze the model's attention focus. The CAM is presented using a heatmap and original image overlay, which helps to understand and analyze the working principles and decision-making process of neural networks (17). The initial learning rate of the model was set to 0.0001, the random seed was set to 1,024, and the epoch was set to 30.

To promote the development of fine-grained computer vision deep learning models, we hosted a congenital heart disease competition on Kaggle (https://www.kaggle.com/competitions/congenital-heart-disease) (18). Every researcher can use the latest models to train and validate on our dataset. It is also essential to provide an interpretable document to help enhance clinical doctors’ pathophysiological understanding.

## Result

### Dataset summary

The dataset includes 828 DICOM chest x-ray files from children, divided into four categories: atrial septal defect (194), ventricular septal defect (210), patent ductus arteriosus (216), and normal control group (208). The distribution of children's gender and age within each group is presented in Table 1.

**Table 1:** Population distribution.

Characteristic	Atrial septal defect	Ventricular septal defect	Patent ductus arteriosus	Normal control group	Total
Number of patients	194	210	216	208	828
Male	83	90	93	89	355
Female	111	120	123	119	473
Age (median)	6.7	6.5	6.8	7.1	6.7

### Model performance evaluation

**Figure 1:**
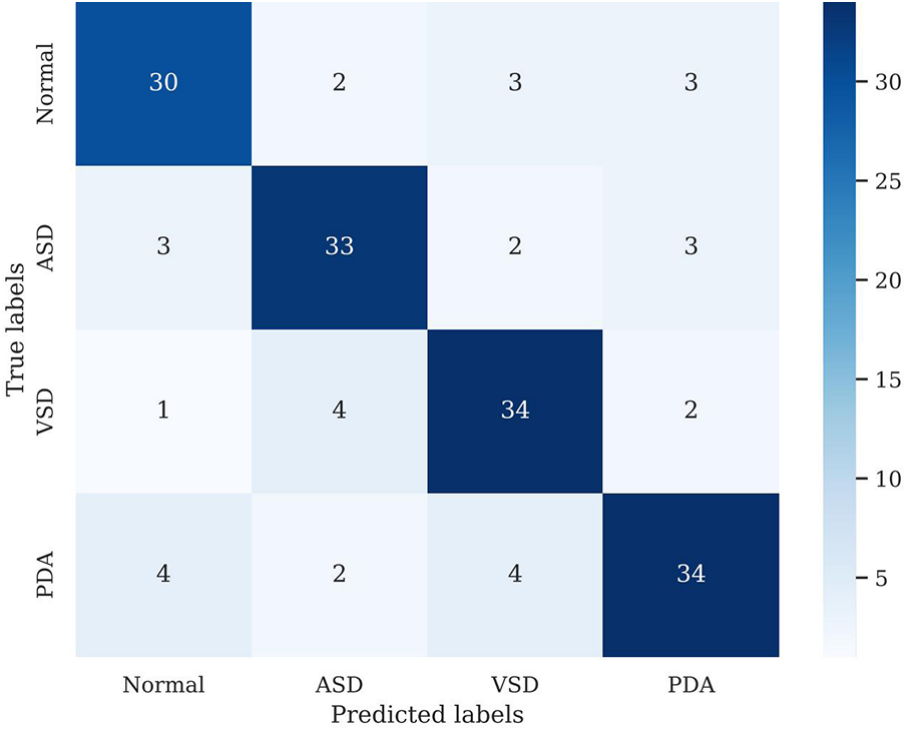
Confusion matrix.

**Figure 2:**
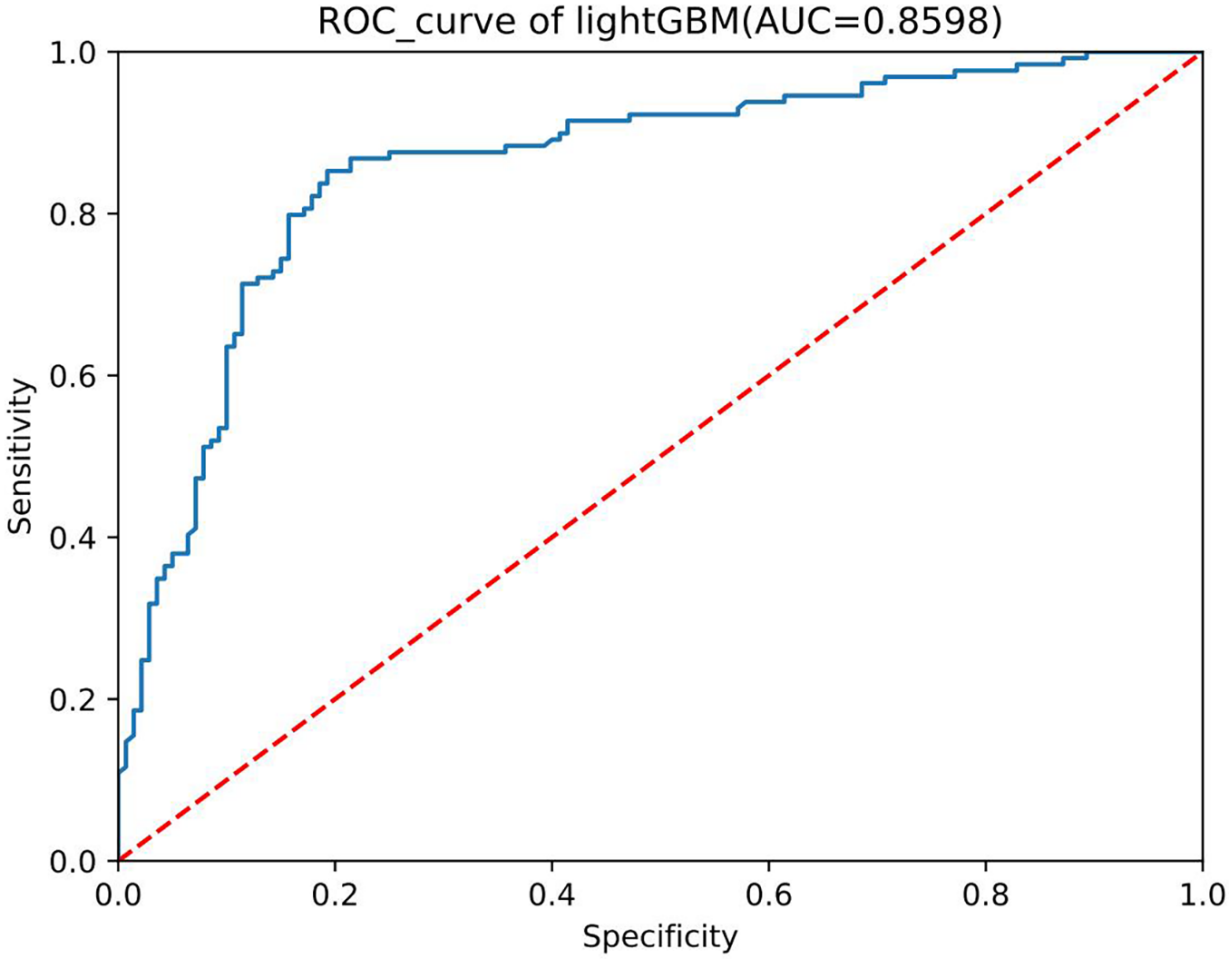
ROC curve.

We conducted preliminary training of the model using the congenital heart disease chest x-ray dataset we constructed. The accuracy of classifying different types of congenital heart diseases reached around 0.8, which demonstrates that our constructed dataset has strong category differences and good representativeness. The prediction results are shown in Table 2. The confusion matrix Predictive accuracy is derived from a confusion matrix (Figure 1). The final ROC curve area under the training model was 0.85, proving that deep learning technology can extract features from fine-grained chest radiographs for classification. The ROC curve of model was shown in Figure 2.

**Table 2:** Model performance.

	Accuracy	Specificity	Accuracy	Recall
Normal	0.7895	0.7913	0.7939	0.7852
ASD	0.8049	0.7982	0.8078	0.7906
VSD	0.7907	0.7842	0.792	0.7826
PDA	0.8095	0.8012	0.7992	0.7946

### Model interpretability assessment

**Figure 3:**
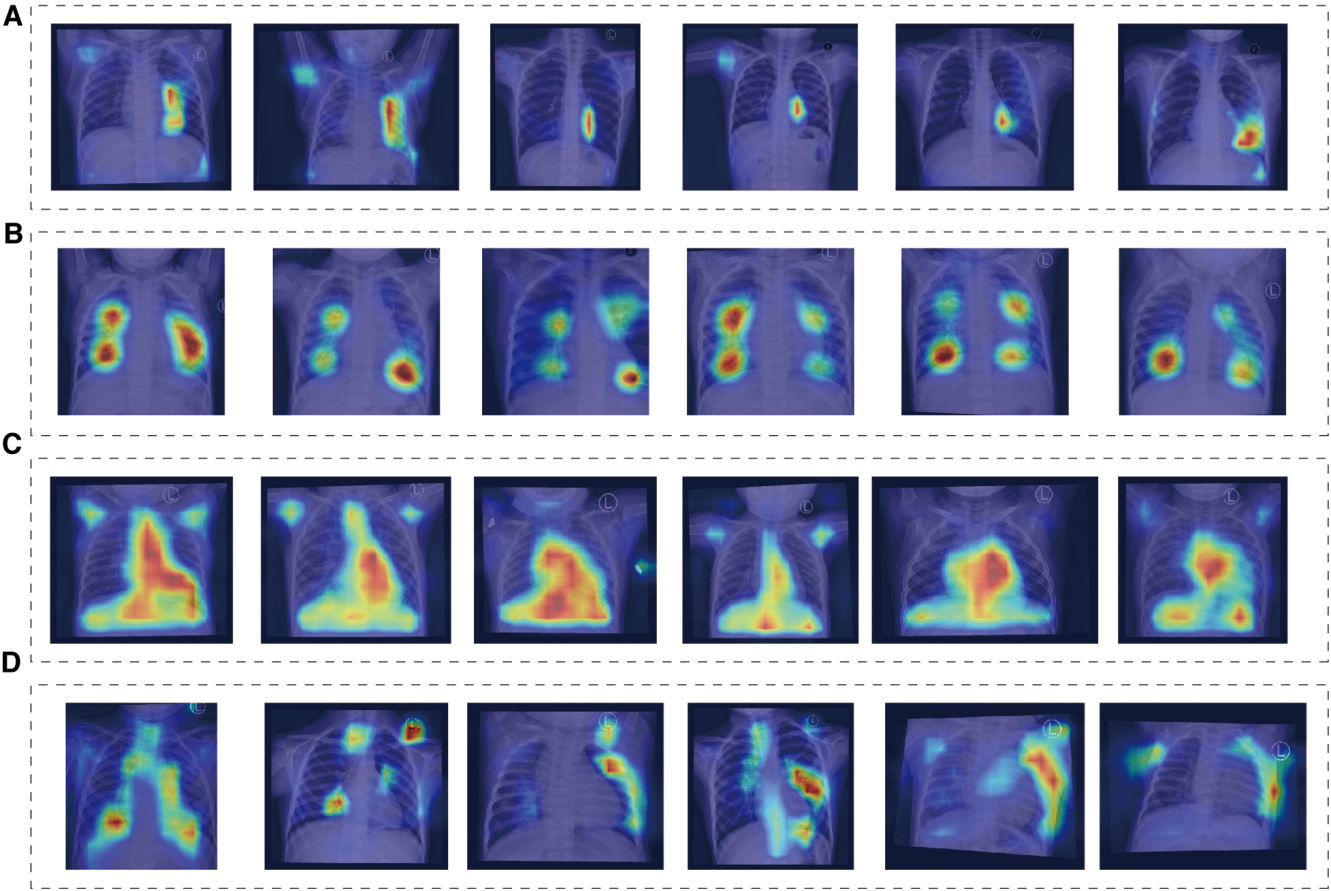
Class activation mapping. (**A**) Normal group, (**B**) ASD group, (**C**) VSD group, (**D**) PDA group.

We found that the main focus was around the heart, and each group showed different attention characteristics, indicating that the model can detect subtle differences that human eyes cannot notice (Figure 3). The distribution of model attention is in line with clinicians' understanding of the pathophysiology of the disease.

## Conclusion

This study constructed the first publicly available deep learning dataset for chest x-ray standardization in congenital heart diseases by collecting chest x-ray images and corresponding clinical information of children with congenital heart diseases. Furthermore, preliminary validation was performed using deep learning algorithms, achieving an accuracy of 0.8 and an ROC of above 0.85. This confirms the availability and representativeness of the dataset.

This dataset serves as a valuable resource for the research community in the field of congenital heart diseases. It not only allows researchers to study the differences in chest x-ray manifestations of various congenital heart diseases but also aids in facilitating early screening of congenital heart disease in underprivileged medical areas. This prompts affected children to seek comprehensive and specialized ultrasound examinations in developed regions.

## Discussion

Our dataset's creation is geared towards enabling the advancement and validation of more precise deep learning algorithms for early detection of congenital heart disease in underserved medical regions. Presently, this dataset stands as the most extensive standardized collection of chest x-rays for congenital heart disease. To assess the dataset's utility, the initial model developed has achieved an ROC area of 0.85, surpassing the diagnostic accuracy of physicians relying solely on chest x-rays. This model can directly aid in the early screening of congenital heart disease in underserved medical areas, facilitating prompt referral and treatment in adequately equipped healthcare facilities.

In this study, we utilized computer vision and machine learning methods, using annotated data, to investigate the classification of chest x-ray examination results in congenital heart diseases. This research aims to enable early screening for congenital heart diseases in children. The potential application areas include analysis, classification, segmentation, and retrieval of images with specific findings or attributes in the field of computer science. Annotated data can also be utilized for medical education and training purposes.

This study advocates for increased researcher participation to propel progress in the field. To foster collaboration and momentum, we have initiated an open Kaggle competition aimed at motivating researchers to further enhance this clinically valuable endeavor. The findings underscore the substantial information within chest x-rays that awaits exploration through more sophisticated deep learning methodologies. Ultimately, this research aims to narrow the gap from machine-learning supported clinicians to machine-educated clinicians.

## Data Availability

The original contributions presented in the study are included in the article/Supplementary Material, further inquiries can be directed to the corresponding author. The DICOM de-identification code was developed using Python 3.11.0 (https://www.python.org/) and Pydicom 1.2.0 (https://pydicom.github.io/). The ResNet18 model was developed using Pytorch (https://pytorch.org/). Applicants are required to accept the terms of data usage and cannot share access to the dataset with others. For publications based on mining this dataset, authors must cite this paper and disclose their code and model. To download and use our dataset, applicants can visit the website (https://www.kaggle.com/competitions/congenital-heart-disease).
